# 2-Chloro-*N*-[(2-methyl­phen­yl)sulfon­yl]acetamide

**DOI:** 10.1107/S1600536811003655

**Published:** 2011-02-02

**Authors:** K. Shakuntala, Sabine Foro, B. Thimme Gowda

**Affiliations:** a Department of Chemistry, Mangalore University, Mangalagangotri 574 199, Mangalore, India; b Institute of Materials Science, Darmstadt University of Technology, Petersenstrasse 23, D-64287 Darmstadt, Germany

## Abstract

In the title compound, C_9_H_10_ClNO_3_S, the amide H atom is *syn* with respect to the *ortho*-methyl group in the benzene ring and the C—S—N—C torsion angle is −66.9 (2)°. An intra­molecular N—H⋯Cl hydrogen bond occurs. The crystal structure features inversion-related dimers linked by pairs of N—H⋯O hydrogen bonds.

## Related literature

For the sulfanilamide moiety in sulfonamide drugs, see; Maren (1976 ▶). For its ability to form hydrogen bonds in the solid state, see; Yang & Guillory (1972 ▶). For hydrogen-bonding preferences of sulfonamides, see; Adsmond & Grant (2001 ▶). For the effect of substituents on the crystal structures of sulfono­amides, see: Gowda *et al.* (2008**a* ▶,b* ▶, 2010 ▶)
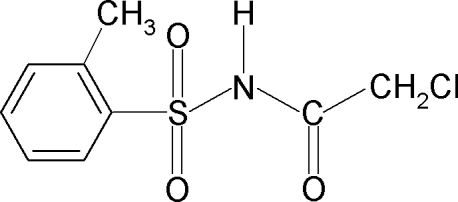


## Experimental

### 

#### Crystal data

C_9_H_10_ClNO_3_S
*M* *_r_* = 247.69
Triclinic, 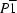

*a* = 7.4439 (8) Å
*b* = 7.5195 (8) Å
*c* = 10.519 (1) Å
α = 93.64 (1)°
β = 109.72 (1)°
γ = 102.52 (1)°
*V* = 535.07 (10) Å^3^
*Z* = 2
Cu *K*α radiation
μ = 4.90 mm^−1^
*T* = 299 K
0.50 × 0.40 × 0.18 mm

#### Data collection

Enraf–Nonius CAD-4 diffractometer
Absorption correction: ψ scan (North *et al.*, 1968 ▶) *T*_min_ = 0.193, *T*_max_ = 0.473
3727 measured reflections
1891 independent reflections
1771 reflections with *I* > 2σ(*I*)
*R*_int_ = 0.051
3 standard reflections every 120 min  intensity decay: 0.5%

#### Refinement

*R*[*F* ^2^ > 2σ(*F* ^2^)] = 0.050
*wR*(*F* ^2^) = 0.137
*S* = 1.08
1891 reflections
141 parameters
1 restraint
H atoms treated by a mixture of independent and constrained refinement
Δρ_max_ = 0.52 e Å^−3^
Δρ_min_ = −0.57 e Å^−3^

### 

Data collection: *CAD-4-PC* (Enraf–Nonius, 1996 ▶); cell refinement: *CAD-4-PC*; data reduction: *REDU4* (Stoe & Cie, 1987 ▶); program(s) used to solve structure: *SHELXS97* (Sheldrick, 2008 ▶); program(s) used to refine structure: *SHELXL97* (Sheldrick, 2008 ▶); molecular graphics: *PLATON* (Spek, 2009 ▶); software used to prepare material for publication: *SHELXL97*.

## Supplementary Material

Crystal structure: contains datablocks I, global. DOI: 10.1107/S1600536811003655/ds2090sup1.cif

Structure factors: contains datablocks I. DOI: 10.1107/S1600536811003655/ds2090Isup2.hkl

Additional supplementary materials:  crystallographic information; 3D view; checkCIF report

## Figures and Tables

**Table 1:** Hydrogen-bond geometry (Å, °)

*D*—H⋯*A*	*D*—H	H⋯*A*	*D*⋯*A*	*D*—H⋯*A*
N1—H1*N*⋯O1^i^	0.79 (2)	2.32 (2)	3.087 (3)	166 (3)
N1—H1*N*⋯Cl1	0.79 (2)	2.62 (3)	2.978 (2)	110 (2)

Symmetry code: (i) 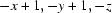.

**Fig. 1.**
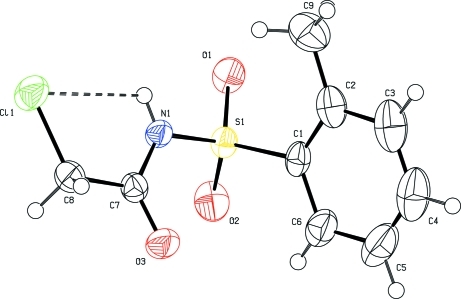
Molecular structure of the title compound, showing the atom- labelling scheme. Displacement ellipsoids are drawn at the 50% probability level.

**Fig. 2.**
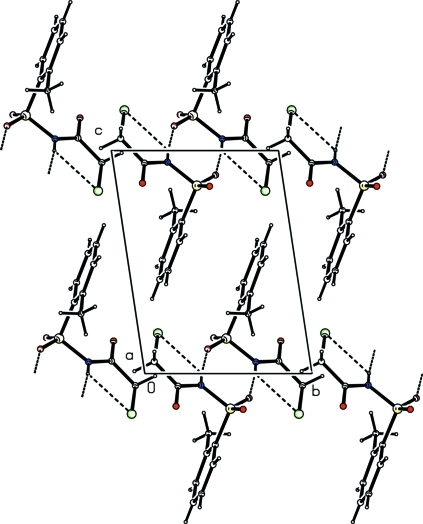
Molecular packing in the title compound. Hydrogen bonds are shown as dashed lines.

## supplementary crystallographic information

### Comment

The molecular structures of sulfonamide drugs contain the sulfanilamide moiety (Maren, 1976). The propensity for hydrogen bonding in the solid state, due to the presence of various hydrogen bond donors and acceptors can give rise to polymorphism (Yang & Guillory, 1972). The hydrogen bonding preferences of sulfonamides has also been investigated (Adsmond & Grant, 2001). The nature and position of substituents play a significant role on the crystal structures of *N*-(aryl)sulfonoamides (Gowda *et al.*, 2008**a*,b*, 2010). As a part of studying the substituent effects on the structures of this class of compounds, the structure of 2-chloro-*N*-(2-methylphenylsulfonyl)- acetamide (I) has been determined. The conformations of the N—H and C=O bonds of the SO_2_—NH—CO—C segment in the structure are anti to each other (Fig. 1), similar to that observed in *N*-(phenylsulfonyl)acetamide (II)(Gowda *et al.*, 2010), *N*-(phenylsulfonyl)- 2,2-dichloroacetamide (III) (Gowda *et al.*, 2008*b*) and *N*-(4-methylphenylsulfonyl)-2,2-dichloroacetamide (IV) (Gowda *et al.*, 2008*a*).

The molecule in (I) is bent at the *S*-atom with a C1—S1—N1—C7 torsion angle of -67.0 (3)°, compared to the values of -58.8 (4)° in (II), -66.3 (3)° in (III) and -71.1 (2)° in (IV). Further, the dihedral angle between the benzene ring and the SO2—NH—CO—C group in (I) is 78.9 (1)°, compared to the values of 89.0 (2)° in (II), 79.8 (1)° in (III) and 81.0 (1)° in (IV),

The structure exhibits both the intramolecular N—H···Cl and the intermolecular N—H···O(S) hydrogen bonds.

In the crystal structure, the pairs of intermolecular N–H···O hydrogen bonds (Table 1) link the molecules through inversion-related dimers into zigzag chains running in the *bc*-plane. Part of the crystal structure is shown in Fig. 2.

### Experimental

The title compound was prepared by refluxing 2-methylbenzenesulfonamide (0.10 mole) with an excess of chloroacetyl chloride (0.20 mole) for about an hour on a water bath. The reaction mixture was cooled and poured into ice cold water. The resulting solid was separated, washed thoroughly with water and dissolved in warm dilute sodium hydrogen carbonate solution. The title compound was reprecipitated by acidifying the filtered solution with glacial acetic acid. It was filtered, dried and recrystallized from ethanol. The purity of the compound was checked by determining its melting point. It was further characterized by recording its infrared spectra.

Prism like colorless single crystals of the title compound used in X-ray diffraction studies were obtained from a slow evaporation of an ethanolic solution of the compound.

### Refinement

The H atom of the NH group was located in a difference map and later restrained to the distance N—H = 0.86 (3) Å. The other H atoms were positioned with idealized geometry using a riding model with C—H = 0.93–0.97 Å. All H atoms were refined with isotropic displacement parameters (set to 1.2 times of the *U*_eq_ of the parent atom).

The U^ij^ components of C3, C4 and C5 were restrained to approximate isotropic behavoir.

### Crystal data

**Table:**

C_9_H_10_ClNO_3_S	*Z* = 2
*M**_r_* = 247.69	*F*(000) = 256
Triclinic, *P*1	*D*_x_ = 1.537 Mg m^−^^3^
Hall symbol: -P 1	Cu *K*α radiation, λ = 1.54180 Å
*a* = 7.4439 (8) Å	Cell parameters from 25 reflections
*b* = 7.5195 (8) Å	θ = 7.0–23.1°
*c* = 10.519 (1) Å	µ = 4.90 mm^−^^1^
α = 93.64 (1)°	*T* = 299 K
β = 109.72 (1)°	Prism, colourless
γ = 102.52 (1)°	0.50 × 0.40 × 0.18 mm
*V* = 535.07 (10) Å^3^	

### Data collection

**Table:**

Enraf–Nonius CAD-4 diffractometer	1771 reflections with *I* > 2σ(*I*)
Radiation source: fine-focus sealed tube	*R*_int_ = 0.051
graphite	θ_max_ = 66.9°, θ_min_ = 4.5°
ω/2θ scans	*h* = −8→8
Absorption correction: ψ scan (North *et al.*, 1968)	*k* = −8→8
*T*_min_ = 0.193, *T*_max_ = 0.473	*l* = −12→12
3727 measured reflections	3 standard reflections every 120 min
1891 independent reflections	intensity decay: 0.5%

### Refinement

**Table:**

Refinement on *F*^2^	Secondary atom site location: difference Fourier map
Least-squares matrix: full	Hydrogen site location: inferred from neighbouring sites
*R*[*F*^2^ > 2σ(*F*^2^)] = 0.050	H atoms treated by a mixture of independent and constrained refinement
*wR*(*F*^2^) = 0.137	*w* = 1/[σ^2^(*F*_o_^2^) + (0.0853*P*)^2^ + 0.2427*P*] where *P* = (*F*_o_^2^ + 2*F*_c_^2^)/3
*S* = 1.08	(Δ/σ)_max_ < 0.001
1891 reflections	Δρ_max_ = 0.52 e Å^−^^3^
141 parameters	Δρ_min_ = −0.57 e Å^−^^3^
1 restraint	Extinction correction: *SHELXL97* (Sheldrick, 2008), Fc^*^=kFc[1+0.001xFc^2^λ^3^/sin(2θ)]^-1/4^
Primary atom site location: structure-invariant direct methods	Extinction coefficient: 0.028 (3)

### Special details

**Table:**

Geometry. All e.s.d.'s (except the e.s.d. in the dihedral angle between two l.s. planes) are estimated using the full covariance matrix. The cell e.s.d.'s are taken into account individually in the estimation of e.s.d.'s in distances, angles and torsion angles; correlations between e.s.d.'s in cell parameters are only used when they are defined by crystal symmetry. An approximate (isotropic) treatment of cell e.s.d.'s is used for estimating e.s.d.'s involving l.s. planes.
Refinement. Refinement of *F*^2^ against ALL reflections. The weighted *R*-factor *wR* and goodness of fit *S* are based on *F*^2^, conventional *R*-factors *R* are based on *F*, with *F* set to zero for negative *F*^2^. The threshold expression of *F*^2^ > σ(*F*^2^) is used only for calculating *R*-factors(gt) *etc*. and is not relevant to the choice of reflections for refinement. *R*-factors based on *F*^2^ are statistically about twice as large as those based on *F*, and *R*- factors based on ALL data will be even larger.

### Fractional atomic coordinates and isotropic or equivalent isotropic displacement parameters (Å^2^)

**Table:**

	*x*	*y*	*z*	*U*_iso_*/*U*_eq_	
C1	0.4813 (4)	0.6426 (3)	0.3198 (2)	0.0356 (6)	
C2	0.6844 (5)	0.7012 (3)	0.3580 (3)	0.0465 (7)	
C3	0.7891 (6)	0.7927 (4)	0.4905 (3)	0.0674 (10)	
H3	0.9259	0.8321	0.5206	0.081*	
C4	0.6944 (8)	0.8259 (5)	0.5778 (3)	0.0764 (13)	
H4	0.7682	0.8892	0.6653	0.092*	
C5	0.4938 (8)	0.7680 (5)	0.5388 (3)	0.0754 (12)	
H5	0.4320	0.7916	0.5992	0.090*	
C6	0.3832 (6)	0.6737 (4)	0.4081 (3)	0.0531 (8)	
H6	0.2468	0.6321	0.3800	0.064*	
C7	0.2227 (4)	0.8123 (3)	0.0563 (2)	0.0336 (5)	
C8	0.1973 (4)	0.9327 (3)	−0.0539 (3)	0.0397 (6)	
H8A	0.2593	1.0596	−0.0110	0.048*	
H8B	0.0574	0.9224	−0.0984	0.048*	
C9	0.7974 (5)	0.6716 (5)	0.2685 (4)	0.0622 (8)	
H9A	0.7241	0.6847	0.1766	0.075*	
H9B	0.8181	0.5500	0.2704	0.075*	
H9C	0.9226	0.7610	0.3010	0.075*	
N1	0.3146 (3)	0.6745 (3)	0.05184 (19)	0.0327 (5)	
H1N	0.366 (4)	0.660 (4)	−0.001 (3)	0.039*	
O1	0.4251 (3)	0.3986 (2)	0.11017 (18)	0.0450 (5)	
O2	0.1386 (3)	0.4445 (3)	0.1603 (2)	0.0516 (5)	
O3	0.1615 (3)	0.8425 (3)	0.14615 (19)	0.0504 (5)	
Cl1	0.29367 (12)	0.88507 (9)	−0.18091 (7)	0.0525 (3)	
S1	0.32891 (8)	0.51904 (7)	0.15752 (5)	0.0320 (3)	

### Atomic displacement parameters (Å^2^)

**Table:**

	*U*^11^	*U*^22^	*U*^33^	*U*^12^	*U*^13^	*U*^23^
C1	0.0487 (16)	0.0278 (10)	0.0267 (11)	0.0120 (10)	0.0075 (10)	0.0051 (8)
C2	0.0512 (18)	0.0318 (12)	0.0458 (14)	0.0066 (11)	0.0055 (12)	0.0113 (10)
C3	0.076 (3)	0.0459 (16)	0.0511 (18)	0.0051 (15)	−0.0078 (16)	0.0068 (13)
C4	0.112 (4)	0.0511 (18)	0.0400 (17)	0.017 (2)	−0.0020 (19)	−0.0004 (13)
C5	0.137 (4)	0.070 (2)	0.0381 (16)	0.051 (2)	0.039 (2)	0.0132 (14)
C6	0.076 (2)	0.0538 (16)	0.0392 (14)	0.0305 (15)	0.0231 (14)	0.0143 (11)
C7	0.0357 (13)	0.0325 (11)	0.0310 (11)	0.0118 (9)	0.0084 (9)	0.0030 (8)
C8	0.0451 (16)	0.0363 (12)	0.0417 (13)	0.0181 (11)	0.0150 (11)	0.0101 (10)
C9	0.0466 (19)	0.0606 (18)	0.076 (2)	0.0087 (14)	0.0209 (15)	0.0130 (15)
N1	0.0407 (12)	0.0335 (10)	0.0287 (9)	0.0161 (8)	0.0141 (8)	0.0063 (8)
O1	0.0661 (14)	0.0323 (9)	0.0412 (9)	0.0231 (9)	0.0185 (9)	0.0057 (7)
O2	0.0432 (12)	0.0498 (11)	0.0537 (11)	−0.0001 (9)	0.0134 (9)	0.0151 (8)
O3	0.0648 (14)	0.0597 (12)	0.0438 (10)	0.0355 (10)	0.0277 (10)	0.0130 (8)
Cl1	0.0744 (6)	0.0547 (5)	0.0462 (4)	0.0325 (4)	0.0314 (4)	0.0216 (3)
S1	0.0384 (4)	0.0259 (4)	0.0298 (4)	0.0087 (3)	0.0093 (3)	0.0049 (2)

### Geometric parameters (Å, °)

**Table:**

C1—C2	1.386 (4)	C7—N1	1.367 (3)
C1—C6	1.398 (4)	C7—C8	1.502 (3)
C1—S1	1.760 (2)	C8—Cl1	1.768 (3)
C2—C3	1.392 (4)	C8—H8A	0.9700
C2—C9	1.494 (5)	C8—H8B	0.9700
C3—C4	1.374 (7)	C9—H9A	0.9600
C3—H3	0.9300	C9—H9B	0.9600
C4—C5	1.367 (6)	C9—H9C	0.9600
C4—H4	0.9300	N1—S1	1.6590 (19)
C5—C6	1.389 (5)	N1—H1N	0.79 (2)
C5—H5	0.9300	O1—S1	1.4303 (18)
C6—H6	0.9300	O2—S1	1.417 (2)
C7—O3	1.208 (3)		
			
C2—C1—C6	122.5 (2)	C7—C8—Cl1	116.35 (17)
C2—C1—S1	122.3 (2)	C7—C8—H8A	108.2
C6—C1—S1	115.3 (2)	Cl1—C8—H8A	108.2
C1—C2—C3	116.8 (3)	C7—C8—H8B	108.2
C1—C2—C9	124.9 (2)	Cl1—C8—H8B	108.2
C3—C2—C9	118.3 (3)	H8A—C8—H8B	107.4
C4—C3—C2	121.3 (4)	C2—C9—H9A	109.5
C4—C3—H3	119.4	C2—C9—H9B	109.5
C2—C3—H3	119.4	H9A—C9—H9B	109.5
C5—C4—C3	121.4 (3)	C2—C9—H9C	109.5
C5—C4—H4	119.3	H9A—C9—H9C	109.5
C3—C4—H4	119.3	H9B—C9—H9C	109.5
C4—C5—C6	119.4 (3)	C7—N1—S1	123.27 (17)
C4—C5—H5	120.3	C7—N1—H1N	124 (2)
C6—C5—H5	120.3	S1—N1—H1N	113 (2)
C5—C6—C1	118.7 (4)	O2—S1—O1	118.71 (12)
C5—C6—H6	120.7	O2—S1—N1	108.92 (11)
C1—C6—H6	120.7	O1—S1—N1	103.64 (10)
O3—C7—N1	122.6 (2)	O2—S1—C1	108.50 (12)
O3—C7—C8	118.3 (2)	O1—S1—C1	110.83 (12)
N1—C7—C8	119.1 (2)	N1—S1—C1	105.34 (10)
			
C6—C1—C2—C3	0.5 (4)	N1—C7—C8—Cl1	−0.7 (3)
S1—C1—C2—C3	−178.1 (2)	O3—C7—N1—S1	7.4 (4)
C6—C1—C2—C9	179.8 (3)	C8—C7—N1—S1	−172.97 (18)
S1—C1—C2—C9	1.2 (4)	C7—N1—S1—O2	49.3 (2)
C1—C2—C3—C4	−1.2 (4)	C7—N1—S1—O1	176.60 (19)
C9—C2—C3—C4	179.4 (3)	C7—N1—S1—C1	−66.9 (2)
C2—C3—C4—C5	1.0 (5)	C2—C1—S1—O2	167.1 (2)
C3—C4—C5—C6	0.0 (5)	C6—C1—S1—O2	−11.5 (2)
C4—C5—C6—C1	−0.7 (5)	C2—C1—S1—O1	35.1 (2)
C2—C1—C6—C5	0.4 (4)	C6—C1—S1—O1	−143.56 (19)
S1—C1—C6—C5	179.1 (2)	C2—C1—S1—N1	−76.3 (2)
O3—C7—C8—Cl1	178.8 (2)	C6—C1—S1—N1	105.0 (2)

### Hydrogen-bond geometry (Å, °)

**Table:**

*D*—H···*A*	*D*—H	H···*A*	*D*···*A*	*D*—H···*A*
N1—H1N···O1^i^	0.79 (2)	2.32 (2)	3.087 (3)	166 (3)
N1—H1N···Cl1	0.79 (2)	2.62 (3)	2.978 (2)	110 (2)

Symmetry codes: (i) −*x*+1, −*y*+1, −*z*.

## References

[bb1] Adsmond, D. A. & Grant, D. J. W. (2001). *J. Pharm. Sci.* **90**, 2058–2077. 10.1002/jps.1157 11745765

[bb2] Enraf–Nonius (1996). *CAD-4-PC* Enraf–Nonius, Delft, The Netherlands.

[bb3] Gowda, B. T., Foro, S., Nirmala, P. G. & Fuess, H. (2010). *Acta Cryst.* E**66**, o1284. 10.1107/S1600536810015849 PMC2979412 21579383

[bb4] Gowda, B. T., Foro, S., Nirmala, P. G., Sowmya, B. P. & Fuess, H. (2008*a*). *Acta Cryst.* E**64**, o1492. 10.1107/S160053680802134X PMC2962122 21203204

[bb5] Gowda, B. T., Foro, S., Nirmala, P. G., Sowmya, B. P. & Fuess, H. (2008*b*). *Acta Cryst.* E**64**, o1522. 10.1107/S1600536808021831 PMC2962148 21203230

[bb6] Maren, T. H. (1976). *Annu. Rev. Pharmacol Toxicol.* **16**, 309–327. 10.1146/annurev.pa.16.040176.001521 59572

[bb7] North, A. C. T., Phillips, D. C. & Mathews, F. S. (1968). *Acta Cryst.* A**24**, 351–359.

[bb8] Sheldrick, G. M. (2008). *Acta Cryst.* A**64**, 112–122. 10.1107/S0108767307043930 18156677

[bb9] Spek, A. L. (2009). *Acta Cryst.* D**65**, 148–155. 10.1107/S090744490804362X PMC2631630 19171970

[bb10] Stoe & Cie (1987). *REDU4* Stoe & Cie GmbH, Darmstadt, Germany.

[bb11] Yang, S. S. & Guillory, J. K. (1972). *J. Pharm. Sci.* **61**, 26–40. 10.1002/jps.2600610104 5066733

